# Retraction: Glucocorticoids Should Be Used With Caution in Patients With SARS-CoV-2

**DOI:** 10.3389/fmed.2022.947016

**Published:** 2022-05-31

**Authors:** Frontiers Editorial Office

The journal retracts the 24 February 2021 article cited above.

Following publication, it was brought to our attention that substantial sections of this article have been previously published. The authors failed to provide a satisfactory explanation during the investigation, which was conducted in accordance with Frontiers' policies.

This retraction was approved by the Chief Editors of Frontiers in Medicine and the Chief Executive Editor of Frontiers. Yong Guo does not agree to the retraction. All other authors agree to this retraction.

